# P02.107. Mindfulness and emotion regulation: the mediating role of coping self-efficacy

**DOI:** 10.1186/1472-6882-12-S1-P163

**Published:** 2012-06-12

**Authors:** C Luberto, S Cotton, A McLeish

**Affiliations:** 1 University of Cincinnati, Cincinnati, USA

## Purpose

Mindfulness, the nonjudgmental awareness of present moment experiences, has received increased scientific attention for its role in promoting mental health. Specifically, mindfulness is associated with fewer difficulties regulating negative emotional states. Recent research has identified mediators of this relationship, including decreased rumination and greater positive emotions. Coping self-efficacy, one’s perceived ability to effectively manage stressors, might be another important mediator, as Eastern philosophy contends that passive rather than active action can enhance a sense of personal control. The purpose of this study was to test whether coping self-efficacy mediated the relationships between specific mindfulness skills and emotion regulation difficulties.

## Methods

Undergraduate participants (N=300; Mage=21.25; 73% female; 83% White) completed questionnaires assessing four mindfulness skills (observing, describing, acting with awareness, accepting without judgment), coping self-efficacy, and emotion regulation difficulties. Pearson correlations were computed for each mindfulness skill and coping self-efficacy, and significant (p<.05) relationships were examined using a series of mediation analyses according to Baron & Kenny.

## Results

Observing was not associated with coping self-efficacy and was dropped from further analyses. Greater accepting without judgment was associated with fewer emotion regulation difficulties (R^2^=.47, β=-.69, p<.001) and greater coping self-efficacy (R^2^=.29, b=.54, p<.01). The effect of accepting without judgment on emotion regulation difficulties dropped when coping self-efficacy was entered into the model (β decreased from -.69 to -.45; total R^2^=.69, p<.001 ). Post-hoc analyses using the Sobel test indicated that coping self-efficacy was a significant mediator (z=-1.62, p<.05). Describing and acting with awareness showed some indirect effect on emotion regulation difficulties through coping self-efficacy, though Sobel tests did not indicate significance.

## Conclusion

Findings suggest that the mindfulness skill of accepting without judgment may improve emotion regulation by promoting self-efficacy for coping with stressors. Future studies should continue to examine other potential mediators to help explain the benefits of mindfulness for emotion regulation.

